# Characterization of the nearly complete mitochondrial genome of ochraceous darkies, *Euphaea ochracea* Selys, 1859 (Odonata: Zygoptera: Euphaeidae) and phylogenetic analysis

**DOI:** 10.1080/23802359.2023.2179355

**Published:** 2023-02-20

**Authors:** Marylin Miga, Puteri Nur Syahzanani Jahari, Sivachandran Parimannan, Heera Rajandas, Muhammad Abu Bakar Latiff, Yap Jing Wei, Mohd Shahir Shamsir, Faezah Mohd Salleh

**Affiliations:** a Department of Biosciences, Faculty of Science, Universiti Teknologi Malaysia, Johor, Malaysia; b Centre of Excellence for Omics-Driven Computational Biodiscovery (COMBio), Faculty of Applied Sciences, AIMST University, Bedong, Kedah, Malaysia; c Environmental Management and Conservation Research Unit (ENCORE), Faculty of Applied Sciences and Technology (FAST), Universiti Tun Hussein Onn Malaysia, Pagoh Higher Education Hub, Muar, Johor, Malaysia; d Centre of Research for Sustainable Uses of Natural Resources (SUNR), Faculty of Applied Sciences and Technology (FAST), Universiti Tun Hussein Onn Malaysia, Pagoh Higher Education Hub, Muar, Johor, Malaysia

**Keywords:** Ochraceous Darkies, Euphaea ochracea, Damselfly, Euphaeidae

## Abstract

In the present study, the nearly complete mitochondrial genome of *Euphaea ochracea* was described and its phylogenetic position in the family Euphaeidae was analyzed. Here, we recovered 13 protein-coding genes, 22 transfer RNAs, 2 ribosomal RNAs and a partial control region, resulting in a mitogenome length of 15,545bp. All protein-coding genes were initiated by the typical ATN codon except *nad3* and *nad1*, which utilizes the TTG codon. Four protein-coding genes (*cox1*, *cox2*, *cox3* and *nad5*) are terminated by an incomplete stop codon T, while others end with either a TAA or TAG codon. The intergenic spacer region, S5, is absent in this mitogenome, supporting the lack of this region as a specific character in damselflies. Phylogenetic analysis showed that the newly sequenced *E. ochracea* is phylogenetically closer to *E. ornata* with a high support value.

## Introduction

**Figure 1.**
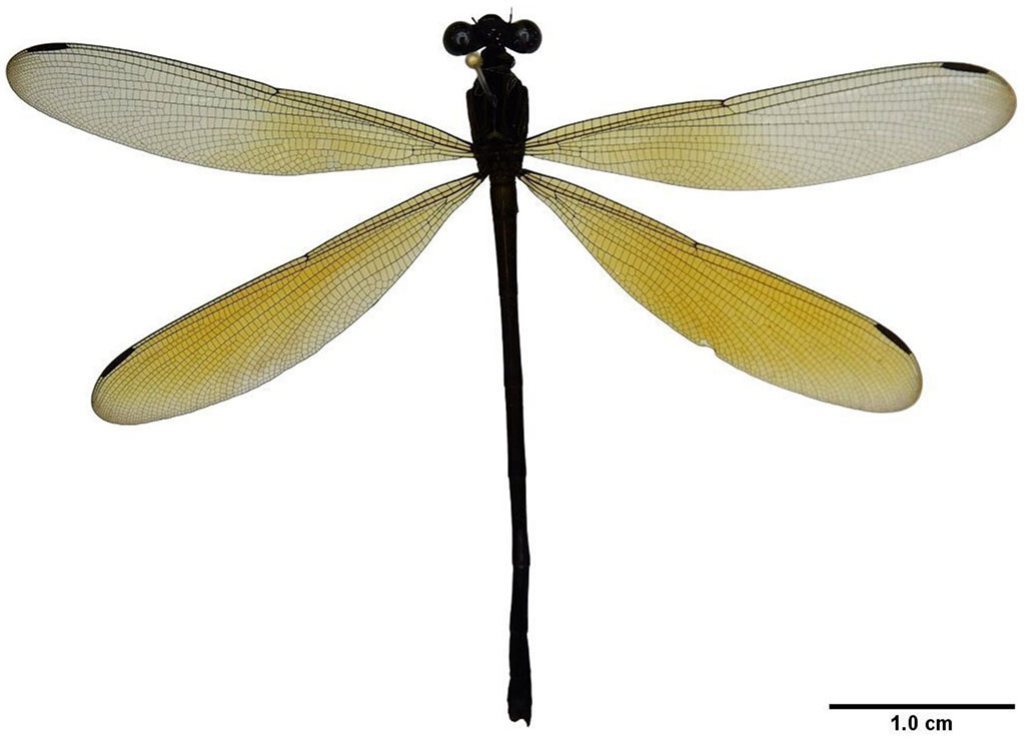
A reference image of *Euphaea ochracea* sequenced in this work, collected from Ayer Hitam Forest Reserve Johor, Malaysia.

Equipped with unparalleled characteristics, Odonata (dragonflies and damselflies) are among the most ancient groups of winged insects and have served as prominent model organisms in the study of insect evolution (Bybee et al. 2016; Jiang et al. 2021). Odonates are also excellent health indicators of aquatic ecosystems and are well-distributed in tropical and subtropical regions (Chee Yen and Mohamed Dawood, 2021). The ochraceous darkies, *Euphaea ochracea* is a damselfly species from the zygopteran family Euphaeidae and often identified based on the golden tip of their wings (male) (Abdul Aziz and Mohamed, 2019) (Figure 1). To date, there are four mitogenomes available for this genus in GenBank, however, the mitogenome data for this species have yet to be reported publicly. Here, we sequenced and characterized a nearly complete mitochondrial genome of *E. ochracea* from Malaysia. The information presented in this work will provide baseline mitogenome data to understand the phylogenetic relationships within Euphaeidae and the study of insect genome evolution.

## Materials

The adult sample of *E. ochracea* was collected from Ayer Hitam Forest Reserve Johor, Malaysia (2.025680, 102.794151) and was identified by a certified taxonomist, Dr. Aqilah Awg Abdul Rahman. The specimen was deposited at Universiti Tun Hussein Onn Malaysia (UTHM) (https://uthm.edu.my; Dr. Aqilah Awg Abdul Rahman; aqilah@uthm.edu.my) with the voucher ID DIO031.

## Methods

### Genomic DNA extraction and sequencing

The total genomic DNA was extracted from the muscle tissue of the hind leg using the Qiagen Blood and Tissue Kit (Qiagen, Valencia, CA), following the manufacturer’s instruction, and fragmented *via* a Bioruptor® system. Library preparation was done using the NEBNext® UltraTM II DNA Library Prep Kit for Illumina® prior to sequencing using the Illumina NovaSeq 6000 (PE150).

### Mitogenome assembly, annotation, and visualization

The raw reads were firstly assessed for its quality with FastQC (https://www.bioinformatics.babraham.ac.uk/projects/fastqc/) and were trimmed for sequencing adapters, as well as low-quality reads using AdapterRemoval (Schubert et al. 2016). With the *cox1* sequence of *E. ochracea* retrieved in the BOLD database (SICO 922-18.COI-5P) as the bait reference sequence, the trimmed reads were then assembled using NOVOPlasty v.4.2 (Dierckxsens et al. 2017). Next, the bwa-samtools pipeline was used to assess the assembled mitogenome by mapping the clean raw DNA reads (adapter free) to the mitogenome sequences. The sequence was then viewed on Tablet (Milne et al. 2009) to manually check for indels and sequence coverage. The nearly complete mitogenome was annotated using MITOS v2 web server (Bernt et al. 2013). The predicted protein-coding genes were further verified with the Open Reading Frame (ORF) Finder server (https://www.ncbi.nlm.nih.gov/orffinder/) following Miga et al. (2022), by aligning the genes with those of the other *Euphaea* species using Jalview 2 v11.1.4 (Waterhouse et al. 2009) to improve the annotation quality. Then, the mitogenome map was produced by Proksee (https://proksee.ca/), an updated version of the CGView web server (Grant and Stothard 2008).

### Phylogenetic analysis

Thirteen protein-coding gene sequences from 10 Odonata mitogenome as representatives were downloaded from GenBank, extracted, and aligned in batches using MAFFT (Katoh et al. 2002) before trimming with Gblocks (Castresana 2000) prior to concatenation. *Ischnura pumilio* (KC878732) (Lorenzo-Carballa et al. 2014) was used as the outgroup and this workflow was performed in PhyloSuite v1.2.2 (Zhang et al. 2020). Phylogenetic analysis was performed based on the Maximum-Likelihood method using the concatenated 13 protein-coding genes in MEGA 11 with 1000 bootstrap values (Tamura et al. 2021).

## Results

**Figure 2.**
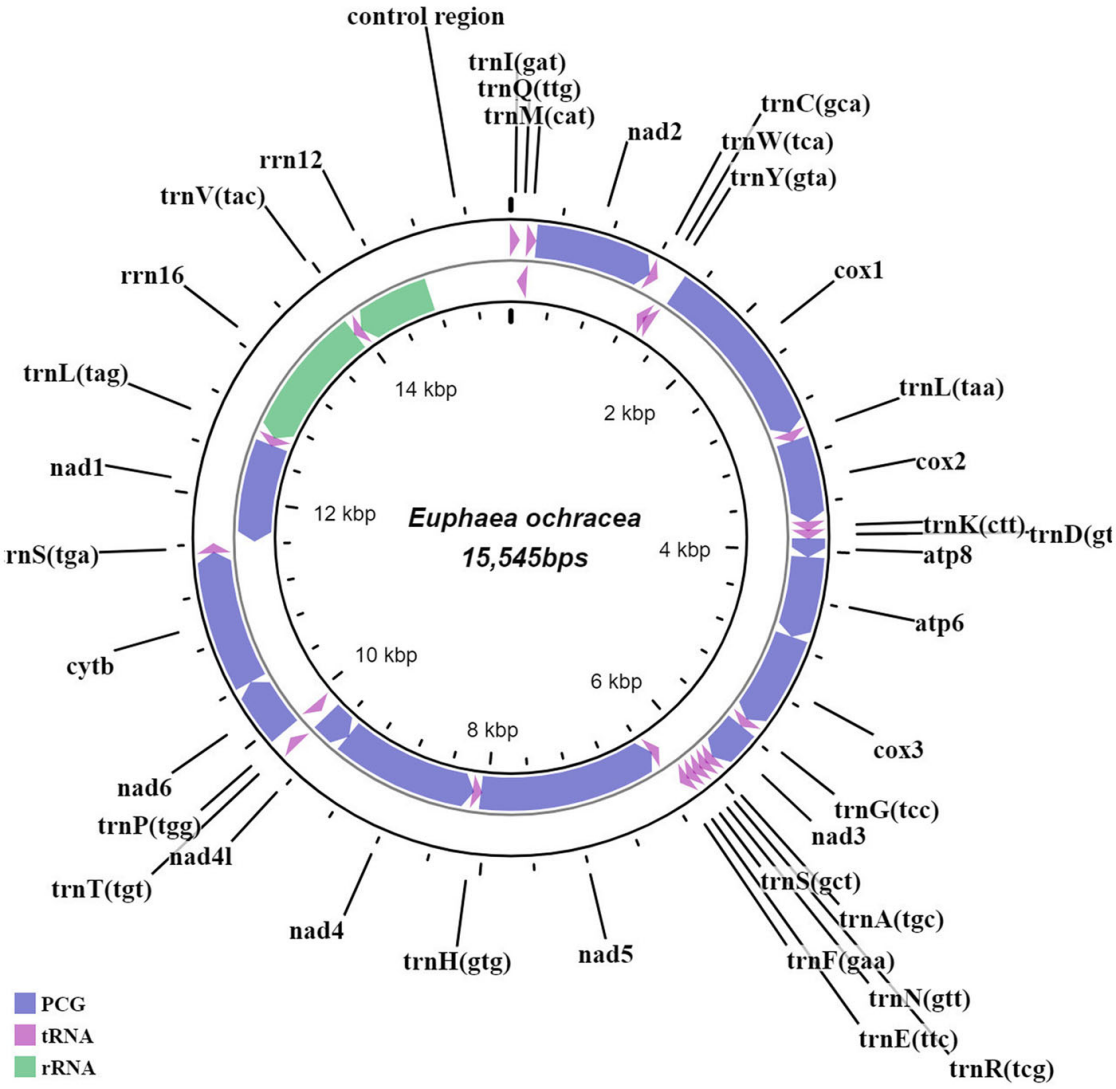
Draft mitogenome map of *E. ochracea* generated in this study. Genes encoded on the heavy strand are transcribed in a clockwise manner, while those encoded on the light strand are transcribed in an anti-clockwise manner. Protein-coding genes are indicated in greyish-blue, transfer RNAs in purple and ribosomal RNAs in green. The innermost circle represents the mitogenome coordinates (Kbp).

The nearly complete mitogenome of *E. ochracea* is 15,545bp in length (GenBank accession no.: ON165247), in which 13 protein-coding genes (PCGs), 22 transfer RNAs (tRNAs), 2 ribosomal RNAs (rRNAs) and partial control region were recovered (Figure 2). The control region which is also known as an AT-rich, the repetitive sequence can be difficult to resolve due to the underrepresentation of AT-rich sequences in Illumina data (Shen et al. 2015). Hence, the *E. ochracea* is presented as a linear mitogenome in GenBank. The mitogenome has a nucleotide composition of A (42.53%), T (28.19%), C (17.05%) and G (12.24%). The nucleotide skew statistics also showed high occurrences of A over T (A–T skew: 0.203) and C over G (G–C skew:0.164). Nine PCGs and 14 tRNAs are encoded on the heavy-strand (H-strand), and the remaining genes are on the light strand (L-strand). The protein-coding gene sequences are 11,145bp in length, while the transfer RNAs are 1,496bp, ranging from 64 bp (tRNA-Cys, tRNA-Arg and tRNA-Ser) to 72 bp (tRNA-Tyr, tRNA-Leu, tRNA-Lys). The 12S and 16S rRNAs are 747 bp and 1,289bp, respectively. Among the 13 PCGs, two PCGs (*nad3* and *nad1*) were initiated by TTG codon, while others utilized the standard ATN start codon. Additionally, incomplete stop codons were observed in four PCGs (*cox1*, *cox2*, *cox3* and *nad5*), while the remaining PCGs were terminated by either TAA or TAG stop codon. In *E. ochracea*, we detected four intergenic spacer regions (s1-s4), consistent with other *Euphaea* species, and short nucleotide overlaps between 8 gene junctions. A BLAST analysis was done on the *cox1* sequence of this species which showed a 98% similarity with the *cox1* sequence of *E. ochracea* from Myanmar (MN345956).

**Figure 3.**
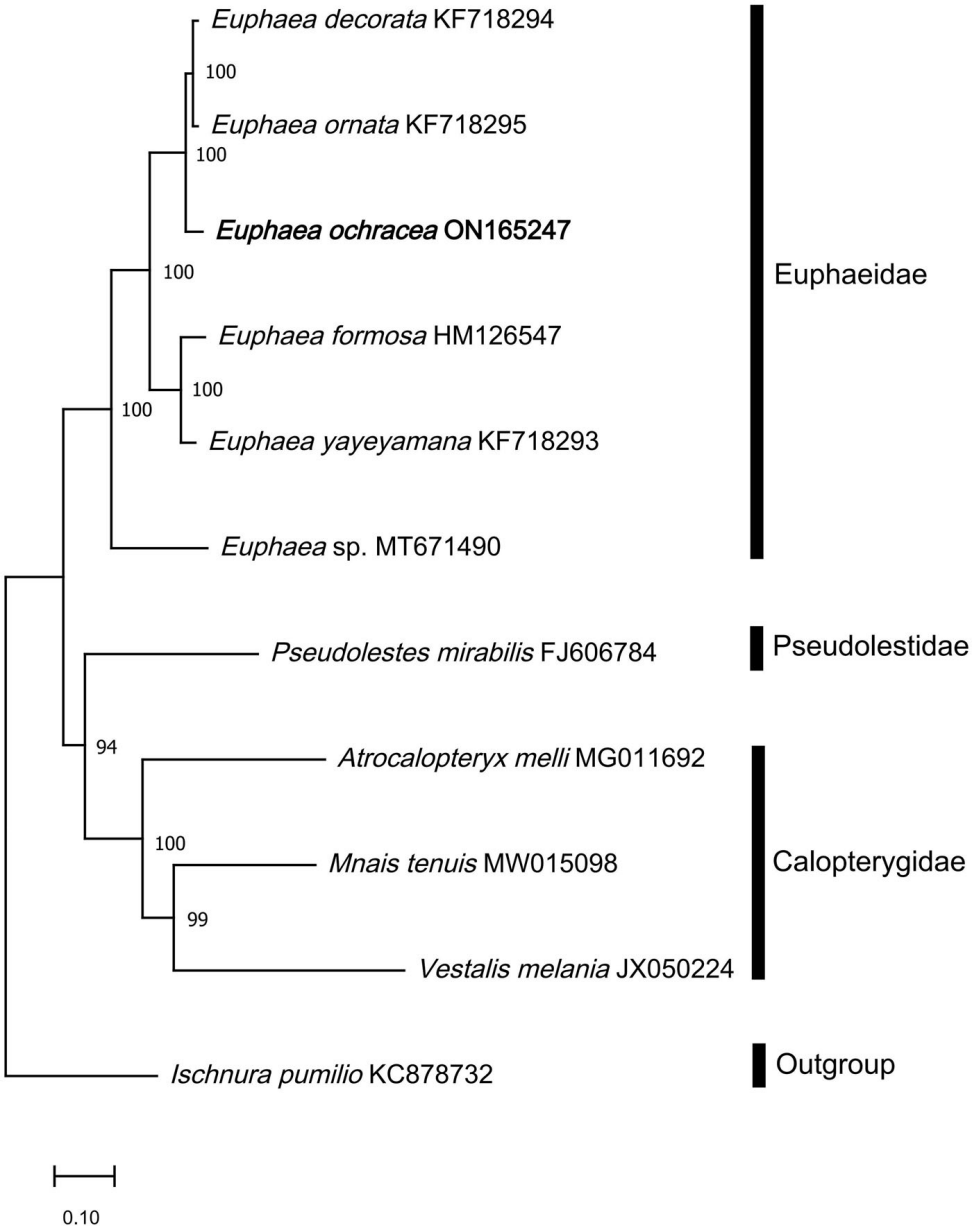
Phylogenetic analysis based on Maximum-Likelihood of 11 Odonata mitogenome sequences, including the newly sequenced *E. ochracea*, using 13 concatenated protein-coding genes (PCGs). All congeneric mitogenome sequences from Euphaeidae was used in the analyses. Nodal support values indicate the Maximum-Likelihood bootstrap support value (BP). The newly sequenced *E. ochracea* is highlighted in bold. *Mnais tenuis* (MW15098) (Wang et al. 2021); *Vestalis Melania* (JX050224) (Chen et al. 2015); *Atrocalopteryx melli* (MG011692) (Xu et al. 2018); *Euphaea decorata* (KF718294), *Euphaea ornata* (KF718295), *Euphaea yayeyamana* (KF718293) (Cheng et al. 2018); *Euphaea* sp. (MT671490) (Macher et al. 2020); *Euphaea ochracea* (ON165247) (this study); *Euphaea formosa* (HM126547) (Lin et al. 2010); *Pesudolestes mirabilis* (FJ606784) (unpublished); *Ischnura pumilio* (KC878732) (Lorenzo-Carballa et al. 2014).

Phylogenetic analysis showed that the majority of the nodes have bootstrap support value higher than 94% (Figure 3). The newly sequenced *E. ochracea* in this study resides together with the other *Euphaea* species in the family Euphaeidae, with a high bootstrap value of 100 and was phylogenetically closer to *E. ornata* (KF718295) (Cheng et al. 2018). Based on the analysis performed here, the family Euphaeidae has a close relationship with the family Pseudolestidae in Zygoptera.

## Discussion and conclusion

This study provided a nearly complete mitogenome of *Euphaea ochracea* and was deposited in GenBank with accession no. ON165247. The nearly complete mitogenome displayed a gene order of tRNA-Ile, tRNA-Gln, and tRNA-Met, which is conserved and identical to other previously sequenced Euphaeidae damselflies. The absence of the s5 intergenic spacer region also supports the lack of this region as a specific character in damselflies (Xu et al. 2018). The phylogenetic relationship of Euphaeidae obtained from the analyses is also consistent with other previous studies, where Euphaeidae is phylogenetically closer to the family Pseudolestidae (Jiang et al. 2021; Wang et al. 2021). The draft mitogenome of *E. ochracea* presented in this work can provide useful DNA molecular data for further phylogenetic and evolutionary analysis in Euphaeidae.

## Data Availability

The genome sequence data that support the findings of this study are openly available in GenBank of NCBI at (https://www.ncbi.nlm.nih.gov/) under accession no. ON165247. The associated BioProject, SRA and BioSample numbers are PRJNA753627, SRR15422665 and SAMN20720553 respectively.

## Permission

This work was done with the permission by the Forestry Department of Peninsular Malaysia under the Research permit JH/100 Jld. 30 (18).
